# Patienthood and participation in the digital era

**DOI:** 10.1177/2055207619845546

**Published:** 2019-04-23

**Authors:** Sonja Erikainen, Martyn Pickersgill, Sarah Cunningham-Burley, Sarah Chan

**Affiliations:** Centre for Biomedicine, Self and Society, Usher Institute of Population Health Sciences and Informatics, University of Edinburgh, Old Medical School, UK

**Keywords:** Patienthood, participation, health consumerism, digital media, social media, big data

## Abstract

The ‘digital era’ of informatics and knowledge integration has changed the roles and experiences of patients, research participants and health consumers. No longer figured (merely) as passive recipients of healthcare services or as beneficiaries of top-down biomedical information, individuals are increasingly seen as active contributors in healthcare and research. They are positioned into multiple roles that are experienced simultaneously by those who access and co-produce digital content that can easily be transformed into data. This is contextualised by ‘big data’ technologies that have altered biomedicine, enabling collation and analysis of myriad data from digitised records to personal mobile data. Social media facilitate new formations of communities and knowledge enacted online, while novel kinds of commercial value emerge from digital networks that enable health data commodification. In this paper, we draw from exemplary digital era shifts towards participatory medicine to cast light on the rapprochements between patienthood, participation and consumption, and we explore how these rapprochements are mediated by, and materialise through, the use of participatory digital technologies and big data. We argue that there is a need to use new conceptual tools that account for the multiple roles and experiences of patient–participant–consumers that co-emerge through digital technologies. We must also ethically re-assess the rights and responsibilities of individuals in the digital era, and the implications of digital era changes for the future of biomedicine and healthcare.

In recent years, scholars across disciplines^1–7^ have documented shifts in the nature of patienthood and participation. These have been propelled by wider socio-technological transformations that have changed biomedical research, healthcare and social interaction in the so-called ‘digital era’ of data analytics, informatics and social media in societies with well-established digital networks. Changes cluster around the intertwined phenomena of ‘big data’ and the increased ‘datafication’ of once-unquantified aspects of life,^3,8^ and the modes of interconnectivity, communication, and information access, sharing and co-production that are enabled by digital platforms. In this context, individuals are continuously and simultaneously positioned as both patients and ‘active’ participants in biomedicine and healthcare by healthcare providers, researchers and research organisations, and policymakers – as well as by themselves and each other. Increasingly, citizens are figured not (just) as passive recipients of healthcare services by healthcare professionals or as mere beneficiaries of top-down biomedical information and health research by researchers, but as active, engaged members in healthcare and biomedical research production. This is exemplified by increasing trends towards what has been called ‘participatory’ medicine and healthcare,^9^ including trends towards personalised medicine and genomics in particular. These aim to provide individuals with personalised biomedical and health information that are both co-produced by and enable individuals to act on and take responsibility for their own health. In the context of increasing commercialisation and globalisation of medical and healthcare services, many people also occupy the role of health consumers in ways that are facilitated by digital health markets and direct-to-consumer (DTC) medicine.^10^ The extent to which these trends are bringing new forms of participation and empowerment at the level of the individual and collectives is, of course, contested. The ways in which patienthood and participation in healthcare and health relevant data production are becoming intertwined and reconfigured demand critical interrogation.

Existing analyses and commentaries have highlighted many potentially empowering as well as dangerous aspects of, and prospects that accompany, the ‘activation’ and ‘responsibilisation’ of patients and citizens in relation to health and medical information in the digital era.^5,6,11^ In this paper, drawing on such scholarship and with examples from our own work, we aim to provide a synoptic account that synthesises the relevant conceptual issues around three key areas: biomedicine and big data; datafication and the construction of community; and commercialising and consuming health. In this way we map how participatory biomedicine and healthcare in the context of social and digital media are blurring and merging the roles and experiences of patients, (research) participants and health consumers. In particular, we examine the seemingly disparate but concurrent move towards population level and ‘personalised’ biomedicine; the processes and practices of (self-)datafication and online health community formation; and the commodification of user-generated content and DTC medicine.

Our aim is not to provide a comprehensive overview of all the diverse emerging digital era formations of patienthood and participation, but rather to draw from exemplary shifts towards participatory medicine to focus on the rapprochements between patienthood, participation and consumption. We explore how these rapprochements are simultaneously mediated by, and materialise through, the use of participatory digital tools and big data biotechnologies, which are reconfiguring the roles of patients, participants and consumers – roles that are often experienced simultaneously by those who access, share and co-produce digital content. Patient–participant–consumers are both subjects and agents of biomedical practices, bringing to the fore that we must not only account for the multiple roles and experiences that co-emerge through digital technologies, but also ethically re-assess the rights and responsibilities of individuals in the digital era. Such interdisciplinary theoretical, methodological and normative tools are required to analyse and appropriately respond to the effects of intersecting and merging digital and biomedical shifts upon citizens as well as healthcare systems and institutions across different contexts. While myriad aspects of these shifts have been explored by others, these analyses have often remained separate, and the intersections of different digital era changes have achieved less attention and scrutiny. The next section provides some wider context before the paper focusses on three key areas of possible transformation.

## Contextualising digital era patienthood and participation

The ‘digital era’ is characterised not only by unprecedented levels of interconnectivity, information access, circulation and co-production, but also by increasing speed, spread and dynamism in relation to knowledge and information turnover. This has been argued to make the information flows of the digital ‘information society’ challenging to manage and control.^12,13^ The current scope of digitally mediated forms of information provision and knowledge management cut across most cultural, social and economic spheres of life.^14^ Indeed, the impact of, and challenges posed by, digital era changes have been analysed in relation to myriad domains ranging from governance and public sector organisation^15^ to human resource management^16^ to marketing^17^ to cultural phenomena like ‘fandom’^18^ in addition to health.^1–7^ The emergence of interactive Web 0.2. digital and social media technologies that enable users to (co-)produce and circulate digital content have facilitated increasing practices of, and platforms for, ‘prosumption’, which refers to the ability of users to both produce and consume information online.^19^ Prosumption has been further enabled by mobile devices and wearable sensors that can simultaneously produce, collate and facilitate access to myriad forms of digital data. The potential and impact of these technologies across sectors and domains have been heralded by technology companies, policy makers and cultural commentators as a ‘digital revolution’.

In the context of biomedicine and healthcare, digital health technologies and applications have been argued to carry revolutionary potential to transform medicine and healthcare in the near future in ways that are promoted as empowering expressions of participatory medicine and ‘citizen science’. Hood et al.^20^, for example, have envisioned that in the next few years, ‘each patient will be associated with a virtual data cloud of billions of data points’ and that these data will be ‘multi-scale’ as well as ‘extremely heterogeneous in type’. They argue that this digitisation or ‘datafication’ of patients will, in turn, facilitate a new model of healthcare that is simultaneously predictive, preventative, personalised and participatory. It will enable patients to benefit from personalised medical information derived from population data stratification, and to take responsibility for managing and improving their own health by monitoring their health data.

In contrast to these promissory visions, others have argued that aspiring towards such ‘predictive, preventative, personalised and participatory’ medicine represents ethically questionable demands for idealistically independent individuality,^21^ medical (self-)surveillance and health-related ‘responsibilisation’, whereby the responsibility for healthcare is increasingly transferred from public and national healthcare services to individuals.^11^ Emphasis on, and celebration of, ‘individual responsibility’ has been argued to reflect neoliberal discourses around decreasing state accountability,^1^ and to mirror Foucauldian modes of power and discipline whereby individuals learn to actively discipline themselves against established norms of health.^22^ While framed as patients’ and citizens’ empowerment, the promotion and use of ‘participatory’ digital health tools and medical self-monitoring technologies can thus be seen as a medicalised form of ‘responsibilisation’.

Simultaneously, the potentials of digital technologies in general and digital health technologies in particular are constrained by inequalities of access and benefit distribution that derive from broader inter- and intra-national socio-economic disparities. A global ‘digital divide’ has materialised along socio-economic lines in multidimensional ways that are characterised by disparities of access to, and use of, digital tools both between high-income and low- and middle-income countries, and between the rich and poor in each country,^23^ as well as along the lines of social dividers such as generational and gendered relations.^24^ In the context of medicine and healthcare, the ‘digital divide’ is intertwined with well-known global health disparities around access to, and quality of, healthcare, which means that inter- and intra-nationally disadvantaged and socio-economically marginalised populations also carry a disproportionate burden of disease.^25^ The ‘digital era’ and its implications for medicine and healthcare have thus not materialised everywhere, and for everyone, to the same degree, which both constrains its healthcare as well as more general potential, and implies that the analysis we advance in this paper is, from the outset, delimited by the scope and reach of digital influence.

Changes in the roles and experiences of ‘patienthood’ and ‘participation’ in the ‘digital era’ are thus intertwined with, and are taking shape in the context of, broader social-economic and geo-political divisions and shifts beyond biomedicine, which in turn are shaping the current bio-technological and healthcare landscape. Our analysis must be located within this wider context, but it also aims to move beyond both the established promises and fears about the potential of the ‘digital era’, by focusing on the nature and implications of mergers between the roles and experiences of ‘patients’, ‘participants’ and health ‘consumers’. These mergers are, potentially, both hopeful and unnerving, but they also carry implications beyond this political and ethical duality.

## Biomedicine and big data

The emergence of digital tools of data collection, collation and analysis has altered the nature and focus of biomedical research and healthcare. The preponderance of so-called ‘big data’, i.e., large quantities of varied, dynamic, easily collated, shared and distributed data^26^, has not only impacted the conduct of biomedical and health research, but also its effects have penetrated into the everyday lives and mundane interactions of patients and citizens more generally, in ways that have implications for how biomedical information is derived and constructed.^24,27^ More generally, digital and quantified health has become a significant dimension of contemporary healthcare and medical practice in many countries. For instance, digital health and medical technologies are being applied not only in biomedical and public health research but also in combination with electronic medical records for the diagnosis, management and treatment of illness and disease.^2^ Ongoing work by one of the present authors explores how this is intertwined with the emergence of new fields such as ‘digital psychiatry’, which has the potential to reshape therapeutic encounters by combining (or dissipating) patient testimonies based on subjective experience with data collected via digital means.^28^ These include so-called eHealth and Health 2.0 technologies and remote access care, as well as various mobile and wearable or implanted devices and biosensors. The use of these technologies in healthcare is producing new bio-techno-social populations.^5,8^ This, in turn, generates new ethical challenges in relation to how digital and social media are being and should be used in healthcare, as well as to find out about, and participate in, ongoing biomedical health-related research. We need to ask how social media is enabling or changing the involvement of patients as participants in relation to health and medical information,^13^ and, importantly, what the ramifications of this are for their care.

Data-driven technologies such as myriad mobile technologies, applications and digital devices, cloud computing, social media and internetworked sensors of various kinds increasingly structure both public and private spheres of life including the workplace, home and leisure activities in addition to healthcare. By using these technologies, we both generate and take advantage of data that are aggregated and analysed by next-generation data analytics.^29^ This includes data from social media platforms, which enable the creation and sharing of user-generated content. By facilitating collection of this generally unsolicited, unmoderated and unsupervised content that can capture details of (qualitative as well as quantitative) information about disease experience that was previously largely outside the purview of medical research, social media are reconfiguring how biomedical knowledge is organised and created.^30^ The production and archiving of patient-generated content and (patient) data such as self-reporting of symptoms and recovery that is shared through social media platforms is increasingly used for medical research in ways that disrupt traditional research modes characterised by organisational settings like hospitals and traditional data collection procedures.^30^ Hence, ‘digitally engaged patients’^2^ become participants in, and co-producers of, biomedical knowledge.^31^ Combined with intertwined phenomena, such as increased networking of previously separate data infrastructures, and the open data movement that promotes open and free access to various kinds of data, big data are producing new data assemblages.^26^ These assemblages are changing how individuals and collective divisions are conceptualised and framed in general and in the context of biomedicine and healthcare in particular.

Big data analytics are designed to extract patterns of information that can be evaluated to construct meaning from the data abundance, such as patterns and differences in disease progression and treatment response within and between patient groups.^32^ The consequent effect of stratifying populations through algorithmically created groupings has enabled new kinds of ‘social sorting’ that are introducing new categories of patients and diseases, at the same time as they can reinforce existing ideas and cultural assumptions about social difference.^29^ Predictive analytics applied to aggregated datasets can produce increasingly powerful evidence of patterns, which are being employed to shed new light on illness and disease risk at population subgroup level. For example, the aggregation of biomedical and population health data with data not conventionally considered ‘medical’ can be applied to identify population level patterns of medically relevant information. Data now considered medically relevant in this context include lifestyle data collected from mobile applications, but also inter alia educational attainment, criminal, income and data collected by government and other public authorities to derive sociodemographic data. New medical populations and subgroups are consequently emerging through big data analytics, based on various previously unknown shared biomarkers and biosocial indicators. The delineation of these subgroups does not occur in a social vacuum: cultural assumptions such as those derived from race, gender and socio-economic inequalities, including presumptions and stereotypes based on social class, may be embedded in the algorithms through which subgroups emerge. Such assumptions can become reinforced in new ways through big data, such as using credit scores to predict and grade health risks including risk of cardiovascular disease.^33^ These phenomena not only signal changing ideas about what counts as medical (or at least medically relevant) data, but also illustrate a broader ongoing shift in biomedical knowledge production away from conventional evidence-based medicine.^26^ While evidence-based medicine has prioritised (time consuming and expensive) hypothesis-driven clinical trials and experimental studies, data-driven approaches and methods are increasingly used to search for associations and correlations within and across datasets, in real time.^34^

With regards to digital curation, patients’ medical information is increasingly collected and stored in electronic health records that together form large databases at a (regional and even national) population level. In Scotland, for example, the Scottish National Health Service and affiliated organisations collect and store population medical records that are analysed to interpret health patterns. Even more recent developments include the Scottish Health Research Register (SHARE) project, which is working to construct a register of the Scottish population that agrees to provide access to their personal medical records for the purposes of further research.^35^ These exist alongside many other similar large-scale population health data endeavours, such as the ‘All of Us’ research programme in the USA to construct an easy-access databank of medical and health data from one million Americans^36^ and whole genome sequencing initiatives like the associated 100,000 Genomes Project in England.^37^ The emergence of digital and big data databanks and research modes positions increasing numbers of patients and citizens as research participants, as we contribute our medical, health and lifestyle data to expanding data archives that are used for biomedical research, population health studies and healthcare interventions. Current and future healthcare is wrapped up in such data initiatives, further blurring the research participant/patient boundary and implying novel responsibilities of such participation.

While, on the one hand, the availability of big health data and big data analysis tools are focusing biomedical and health research attention towards population level research, on the other hand, research that applies population level data is increasingly aimed at translating such data into personalised or individualised treatment solutions.^38^ This creates a sphere of interaction in which health is simultaneously increasingly individualised yet understood through the lens of populations. Digita and technology-based research tools and methods, such as genomics and other so-called ‘-omics’ fields, such as proteomics aimed at the collective characterisation of proteins, and systems-level modelling of biomedical data are increasingly hoped by biomedical, industry and governmental commentators to enable better targeted and more *personalised* treatments.^39^ Resonant with shifts documented following the rise of such genomic technologies in public health, digitisation enables the direction of ‘scientific, biomedical, and public health attention both *inward* […] and *outward*’.^40^

## Datafication and the construction of community

Practices of self-‘datafication’, most notably what is often termed self-tracking and the ‘quantified self’ movement, are increasingly promoted by companies and media commentators, as well as engaged in by a variety of publics. These forms of voluntary self-monitoring or surveillance feature the recording and quantification of various features of everyday lives.^41^ In turn, these act as the basis for constructing digital communities of self-trackers, within which one’s own data can be shared and (re)interpreted through the lens of data generated by others.^41,42^ These practices, and the technologies that enable them, enact new representations and conceptualisations of the self in relation to others, producing detailed biometrics of the body, behaviour, lifestyle and environment that deconstruct and reconstruct bodies and subjectivities in quantitative terms. This process is especially visible in the domain of health and wellness, where one’s nutritional, fitness and other lifestyle data can be monitored, then compared to a quantified health ‘norm’ that is created by the aggregation of large quantities of lifestyle data from others, and ultimately used to construct self-knowledge that can be applied by individuals to modify their lifestyle practices and ‘take responsibility’ for (or responsibilise) their own health.^41,43^

Simultaneously, digital and social media have innovated patient options for interacting, generating networks, forming identities and communities, and mobilising in unprecedented ways – as well as accessing cutting-edge information about biomedical science and technology, healthcare law and practice. Specialised platforms such as *PatientsLikeMe* – and general platforms like Facebook, Twitter and YouTube – participate in the production of patient experiences. In particular, they enable the creation and sharing of, and access to, content produced by patients about their experiences of disease, illness, treatment and recovery. By aggregating individual experiences, these platforms function as databases of experience^2^ which include advice and recommendations about treatment options and forms of care that patients and prospective patients can use as a resource in health-related decision-making. Digital patient communities and spaces enable patients to re-frame and challenge information and messages distributed by competing organisations and agendas, including officially authorised stories, to produce counter-hegemonic discourses of their own that function as alternative knowledges.^44^ The discourses and frames that individual patients are faced with will, in turn, be influenced by the social networks to which they belong. Digital networks are not neutral spaces: existing connections, as well as links enabled and forged by the structure of the social media platform itself, influence which information and messages users encounter and which interpretations they elect to make.

This circulation of digital and social media-facilitated information is also connected with the broader shifts in biomedical knowledge production around which kinds of information can count as ‘evidence’, not only in relation to the harnessing and use of digital and social media data in biomedical research, but also with regards to increasing disillusionment among some publics with conventional medicine and medical research. This is particularly evident when it comes to controversial experimental and alternative forms of treatment and medical intervention that are marketed directly to consumers online, such as so-called ‘unproven’ DTC stem cell therapies that do not conform to the standards of evidence-based medicine, including clinical trials.^45–47^ Information distributed by DTC clinics offering these therapies as well as user-generated content on social media provide alternative sources of information, in addition to (or instead of) conventional medical sources. This is especially pertinent when patients’ priorities do not align with the priorities of the medical and healthcare professionals who treat them. Exemplary is the ‘right to try’ movement, which is focused on patients’ access to experimental therapies and drugs. The movement has unfolded at the intersection of patients’ priorities and need for treatments for diseases for which no evidence-based treatments exist – including conventionally disregarded emotional needs such as the value of hope^44,48^ – and the priorities and concerns of medical and research professionals working within evidence-based epistemic frames.^49^ Their priorities are focused on effectiveness, including cost effectiveness, and safety of medical care, even when compassionate use and experimental intervention remain part of the medical framework.

In addition to community formation around specific disease categories, new participatory health communities are consolidating through the populations produced by the use of big data technologies and analytics in biomedicine, facilitated by social media. These include groups of individuals identified through screening technologies as being ‘at risk’ of a disease, who can then revise their identity conceptions in relation to the ‘at risk’ category. They can also act on information about how to manage health under the condition of being ‘at risk’, in ways that shape experiences and identity formation in relation to health and illness even in the absence of a disease diagnosis.^50^ Ongoing work by one of the authors with colleagues^51^ explores how social media are facilitating the formation of collective identities and digital communities through shared experiences of being ‘at risk’ of cancer due to inherited genetic syndromes. For individuals ‘at risk’, social media such as online blogging enable not only the formation of social connections around their specific type of cancer risk,^52^ but also around experiences shared with those with other inherited syndromes. This, in turn, facilitates the construction of networks around the notion of risk (and new fora for interpreting and negotiating this notoriously slippery concept).

Digitally mediated network formation around these emergent medical classifications illustrates how digital and big data biomedical research technologies and digital and social media platforms can co-create new forms of experiencing and interpreting the meaning of patienthood, as well as participation in medical care and (self- or risk-) monitoring. These include experiences of patienthood ‘in waiting’^51^ that come to be constituted in relation to risk of disease. The centring of ‘risk’ in these ways highlights that contemporary experiences of patienthood are shaped not only by the digital era, but also by (related) socio-political changes in how ‘risk’ is conceptualised and managed. Beck^53^ among others has argued that we are increasingly living in ‘risk societies’, where omnipresent and myriad forms of ‘risk’ (from environmental to terrorism to health) dominate public and political discourses. In ‘risk societies’, ignorance or indifference towards these risks is discursively positioned as a threatening failure to anticipate and mitigate them: ‘not knowing’ or preparing for them gives rise to disaster, while knowing and predicting enables risk mitigation and control.^54^ In the context of health, emphasis on the management and control of risks is exemplified by public health governance shifts towards ‘responsibilisation’ and individual accountability over health, as well as by increasing interest and investment in ‘preventative’ healthcare and the use of predictive analytics in biomedical research.^55^

## Commercialising and consuming health

In addition to carrying research and community value, patient generated digital data have increasing commercial uses. Social media data can be collected and commodified by the platform developers for profit, including the on-selling of the data to clients in the commercial and private healthcare sector.^31^ Such economic practices give rise to complex ethical issues including around ownership and quality of research, control of health data and data infrastructures, and new power asymmetries around data access.^56^ The new and emerging modes of information access, sharing and production as well as the kinds of value created inextricably intertwine with the forms of communication and interconnectivity enabled by the digital era.

Efforts to attach commercial value to and profit from health data have encouraged the emergence of new relations that are increasingly blurring the boundaries of medicine, healthcare and commerce.^29^ For example, communication and IT companies such as Vodafone, Apple and IBM have been working to connect payers, healthcare providers and product innovators by creating digital ecosystems that capitalise on the self-tracking and internet-of-things movements. These companies can conduct analytics of their own through these ecosystems, and repackage and on-sell the data as a product. Similarly, global management consulting companies such as KPMG are investing in health analytics, and applying their expertise in data analytics in relation to ‘therapeutic value optimisation’ such as assessing and demonstrating a drug’s value as well as tracking health outcomes.^29^ The entry of these new participants into healthcare systems is re-routing traditional relations of information flow between payers, service providers, physicians and patients, as well as blurring the lines between the public and private, and medical and non-medical domains.^29^

These digital ecosystems, cross-spatial interconnectivity and forms of communication are connected with the emergence of new global healthcare markets which simultaneously position individuals not only as patients and research participants, but also as (health) consumers. DTC marketing, often online, of experimental therapies such as stem cell treatments taps into and capitalises on patients’ need for hope and demands for treatment despite lack of evidence for the treatments’ effectiveness and safety.^45^ This can also render patients as paying participants in the construction of an evidence base for the effectiveness of experimental medical interventions in the absence of clinical trials.

DTC marketing of biomedical technologies also builds on broader discourses around patient and consumer empowerment, at the same time as pushing towards individuals taking greater responsibility for their own health. Such marketing has co-occurred with the drive towards personalised medicine that has been catalysed by advances in genomics and reorientations of healthcare systems internationally.^57^ For example, companies offering DTC genetic testing for disease susceptibility appeal to the importance of self-knowledge in relation to one’s personal genetic profile and related disease risk factors, often without the direct involvement of healthcare professionals in interpreting the testing results.^58^ The marketing of these services mobilises claims to provide consumers with a map of genetic risk factors that can be used to modify lifestyle behaviours in ways that enable individuals to improve their health.^10,59^ Notably, genetic testing companies such as 23andMe have actively fostered consumer communities through various online fora, including social media platforms like Twitter and Facebook.

The rhetoric of empowerment is, however, intertwined with ‘responsibilisation’, as taking greater responsibility for one’s own health can easily translate into increased expectations or obligations to maintain and promote personal health, in ways that can transfer responsibility away from publicly funded healthcare organisations, the modification of environmental influences on health and towards individuals. Simultaneously, consumers are increasingly being positioned as ‘active’, not only in terms of health seeking, but also in terms of having control over how their data are used in emerging regulatory and cultural environments. In particular, the new EU-wide General Data Protection Regulation aims to reinforce data subjects’ rights to privacy and protection of personal data in digitised environments in ways that have particular implications for ‘special’ (i.e. sensitive) categories of data, such as health data.^60^

Indeed, the use of consumers’ and citizens’ health data for commercial as well as for public health promotion purposes raises questions about data ownership, use and access, as well as about possible data breaches and the harms that may consequently result. For example, data breaches in relation to eHealth monitoring technologies and applications – including fitness and nutrition tracking but also disease symptom, sexual activity and mental health tracking, among other sensitive information – could carry serious adverse implications if the data could be traced back to those from whom it was collected.^27^ The possibility of sharing these kinds of data with healthcare providers also raises questions about the extent to which such data could be used in treatment decision-making to ensure compliance with behavioural regimes that have been medically recommended, such as levels of physical activity or food consumption.^27^ These questions are especially pertinent in relation to socially stigmatised groups such as those defined as overweight or obese; stigmatised illnesses such as many mental health conditions and sexually transmitted diseases; and stigmatised behaviours such as sexual activities that fall outside social norms.

Additionally, commercialised practices such as DTC medicine also have the capacity to shape and reconfigure the process of identity formation. In relation to DTC genetic testing, for example, ‘knowing’ one’s genetic and genomic constitution can enable the reinterpretation of one’s biogenetic–biographical positionality. Companies offering genetic testing services, which have carried names such as ‘Knome’ and ‘deCODEme’, make explicit claims to the centrality of genomic data in relation to the constitution of the self, and many of these companies offer services such as ancestry tracing – including information pertaining to socially significant designators like ethnicity – in addition to disease susceptibility testing.^61^ Genomic data in general, and commercial DTC genetic testing in particular, have enabled the formation of new medical categories and forged communities and ‘biosocial’ networks built on shared experiences of making sense of genomic susceptibility data, such as communities of individuals ‘at risk’ or ‘patients in waiting’.^62^ At the same time, technologies like amniocentesis enable people to entwine their cultural notions of parenthood and family with genetic knowledge in ways that are re-fashioning the culture and politics of reproduction.^63^ These developments have complex implications for how social categories, identities and relationships including ethnicity and kinship are understood, and how biographical narratives are constructed.^61^

## Conclusion

So-called participatory modes of biomedicine and healthcare in the context of the ‘digital era’ of social and digital media – and the heterogeneous identity and (bio)social network formations and possibilities for agency that are enabled by, and enacted through, digital tools – are reconfiguring and blurring the roles and experiences of patienthood, participation, and health and medical consumerism.^4,7,9,31^ These roles and experiences are layered concurrently, and in new ways, onto those who access, share and (co-)produce content that can easily become data in semi-public digital spaces. Together with big data informatics, analytics, related new biomedical research modes and the ‘datafication’ of life, participatory digital era health biotechnologies shape the constitution of new, often cross-border communities, social movements, epistemologies, knowledge practices and health markets,^44,50,51^ while excluding others from such activities.^23,24^

Big data biotechnologies are facilitating the collection, collation and analysis of unprecedented quantities of heterogeneous data on a population scale, at the same time as the large databases that result from this enable population subgroup stratification. This, in turn, is motivating a seemingly incompatible yet concurrent shift towards ‘personalised’ medicine and healthcare.^21^ The ‘datafication’ of citizens’ and patients’ experiences, embodiments and information more generally are reconfiguring how the self can be and is conceptualised, and how medical knowledge can be and is produced by researchers and healthcare professionals as well as by citizens and patients themselves.^3,29,31^ The forms of communication and interconnectivity facilitated by digital and social media platforms simultaneously turn individuals into digital subjects and shape the forms of identity, community and knowledge that are produced and circulate there.^44,61^ New kinds of commercial value in turn emerge from and through the data abundance and digital networks that enable medical and health data to be harnessed for profit, as well as commodified and marketed directly to consumers.^31,45,46,58,59^

These intertwined phenomena have arisen and are taking shape through and in response to the ‘digital era’, and they underscore the evolving and increasingly tangled roles of patients, participants and health consumers as both subjects and agents of science and healthcare. Such entanglements are, however, shaped by the overarching social, economic and political contexts in which they are embedded, as well as by pre-existing social and cultural disparities and inequities. This implies that the empowering as well as problematic potentials of digital era developments are both constrained and facilitated by concurrent socio-political movements. They are also constrained by modes of social and economic stratification including classed, racialised and gendered divisions that pre-date the digital era. Indeed, the ‘digital era’ itself has not materialised in the same way across contexts, but is unevenly inter- and intra-nationally distributed along the lines of broader global geo-political and socio-economic as well as public health disparities.^23^ This brings to the fore the importance of continuous ethical, social and political scrutiny of the proliferation of rights and responsibilities attached to individuals, groups and organisations in relation to new and emerging digital biotechnologies and participatory biomedicine. In particular, attention needs to be directed towards the rights and responsibilities of, as well as benefits and harms to, those living and working beyond the university and healthcare sectors who nevertheless shape the production of biomedical and health-related knowledge in important (and sometimes under-appreciated) ways. Normative work, including by one of the authors of this paper,^27^ has already begun for developing frameworks that appropriately account for the roles of these actors.^21,56,63,64^

There is, then, a need to work with fresh conceptual tools for understanding the digitally mediated entanglements of patienthood, participation and health consumption, as well as the effects and consequences of these entanglements for healthcare practices, biomedical and health research and innovation. These tools need to be tuned to address the intersection of big data, ‘datafication’, emerging participatory medical and healthcare modes, and related research innovation, digital globalisation and global disparities, and new forms of interaction including social media community and identity formation. New social, ethical and legal frameworks and solutions will require engaging across disciplines, contexts and sectors; sharing and debating widely and inclusively.^7^ This work is required, not only to understand how the digital era is changing the roles and practices of patient–participant–consumer, and the consequences of these changes for future health challenges, but also to intervene in ways that support patient autonomy and improvements in health and well-being.
